# How to make hand sanitiser/hand rub

**Published:** 2020-09-01

**Authors:** Abeer HA Mohamed Ahmed, Choon Fu Goh

**Affiliations:** 1 Research Fellow in Pharmacology and Clinical Trials, Pharmacist: London School of Hygiene & Tropical Medicine, London, UK.; 2 Senior Lecturer in Pharmaceutical Technology/ Leading Researcher in Skin Research Group: School of Pharmaceutical Sciences, Universiti Sains Malaysia, Minden, Penang, Malaysia.

**Hand sanitisers (also known as hand rub) and hand washing play important roles in fighting viral infections.**

The COVID-19 virus can be transmitted when someone touches a contaminated surface and then touches their mouth, nose or eyes, and contaminated hands can also transfer the virus to other surfaces.

The World Health Organization (WHO) recommends hand washing with soap and water for 20 seconds to prevent contact transmission. WHO also recommends the use of alcohol-based hand sanitisers based on the following factors^1^, ^2^:

- They are effective in killing microorganisms
- They are suitable for use in resource-limited or remote areas with lack of accessibility to clean water and sinks
- Hand hygiene using hand sanitiser is easy, fast, and accessible at the point of patient care
- It is affordable to make
- There are few adverse effects.

Alcohol is the active ingredient in hand sanitisers. At high enough concentrations, it will destroy most viruses, bacteria and fungi by denaturing (changing the shape of) the proteins that make up these microbes.

For hand sanitiser to be effective, the final formulation should be 80% ethanol or 75% isopropyl alcohol. To achieve that concentration, the instructions below require either:

- Ethanol (96% alcohol), or
- Isopropyl alcohol (99.8% alcohol).

**Note:** The World Health Organization recommends that hand sanitisers are used on skin with **no visible dirt**. If your hands are visibly dirty, wash them with soap and water (Figure 1).

**Figure 1:**
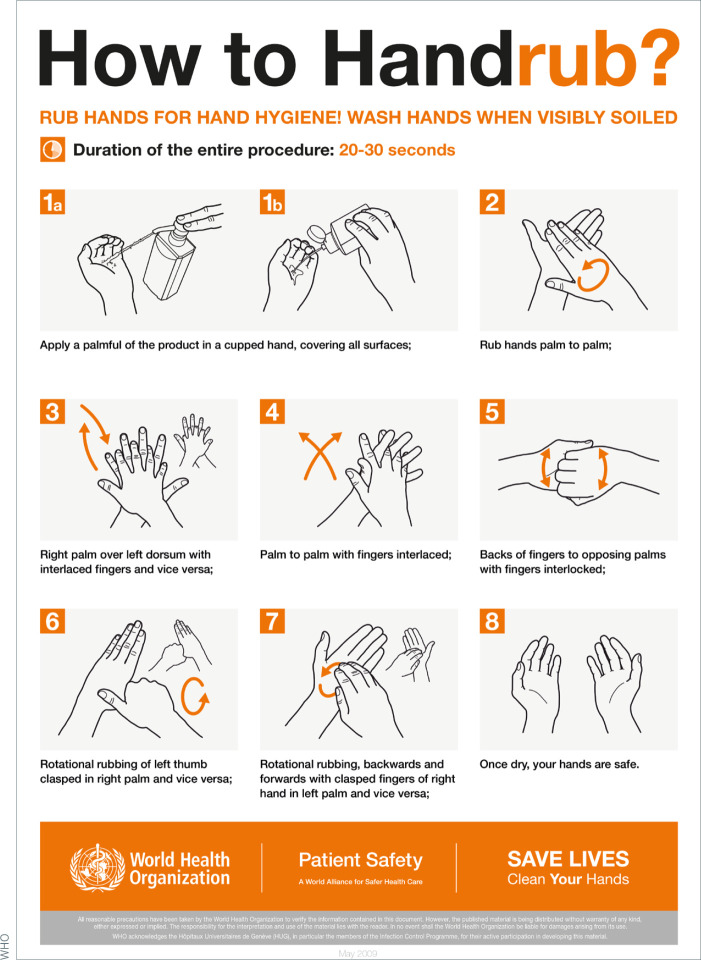
World Health Organization poster on how to use hand sanitiser

## What you will need

To make 10 litres of hand sanitiser, you will need:

### Ingredients

- Alcohol: 8,333 ml (millilitres) ethanol 96%, or 7,515 ml isopropyl alcohol 99.8%
- Hydrogen peroxide 3% (417 ml)
- Emollient: glycerol (glycerine/glycerin) 98% (145 ml)
- Sterile distilled or cooled boiled water (just over 1.1 litres when using ethanol, and just over 1.9 litres when using isopropyl alcohol)

### Equipment

- Container for mixing: a large, clean container or ‘tank’ with a minimum volume of 10 litres, with a cap or screw top (and made of glass or translucent plastic so you can see the liquid level)
- Measuring cylinders and measuring jugs
- A plastic or metal funnel
- 100 ml plastic bottles with leak-proof tops and/or 500 ml glass or plastic bottles with screw tops for distributing the hand sanitiser to handwashing stations or individual health care workers

## Procedure

**Figure 2:**
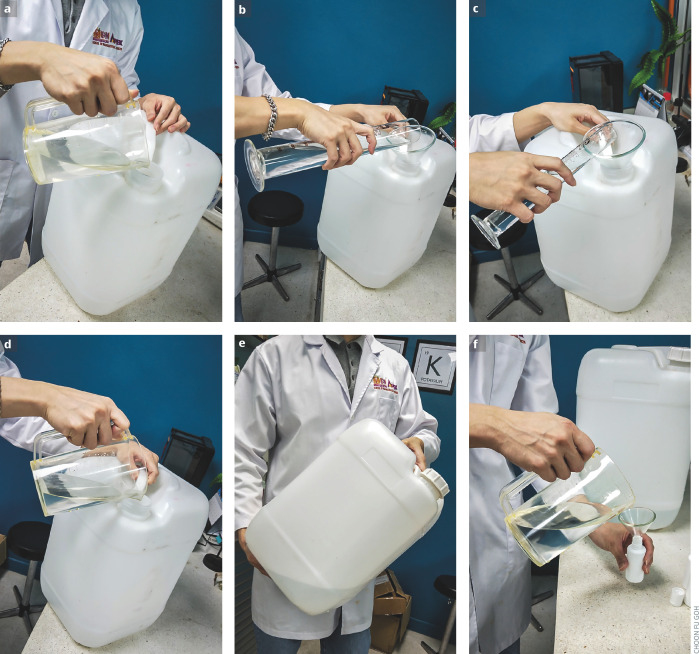
Preparation of alcohol-based hand sanitisers according to WHO guidelines^1^

- Clean the working surfaces.
- Wash your hands and put on a clean lab coat or an apron.
- Gather the ingredients and place within easy reach.
- Measure and mark the 10-litre level on the outside of the mixing container.
- Place the funnel in the opening of the mixing container.
- Use the measuring jug and/or cylinder to measure and pour the alcohol (8,333 ml of ethanol 96% or 7,515 ml of isopropyl alcohol 99.8%) into the mixing container (Figure 2a).
- Measure 417 ml of hydrogen peroxide using a measuring cylinder and add to the mixing container (Figure 2b).
- Measure 145 ml of glycerol using a measuring cylinder and pour it into the mixing container (Figure 2c).
- Glycerol is very viscous and will stick to the wall of the measuring cylinder; therefore, rinse the cylinder with some of the sterile distilled or cooled boiled water and empty this into the mixing container.
- Add sterile distilled or cold boiled water into the bottle or tank to the 10-litre mark (Figure 2d).
- As soon as possible after all the components have been added, firmly close the mixing container to prevent evaporation.
- Shake the mixing container gently to mix the solution (Figure 2e).
- Pour the solution into the dispensing bottles, e.g. 500 ml or 100 ml glass or plastic bottles (Figure 2f).
- Store the bottles for 72 hours before use to make sure that any microbes that may have been present in the mixing container or the new/reused bottles are destroyed.
- Label the bottles with the final concentrations of ingredients (Table 1).

**Table 1:** Final concentrations of ingredients in hand sanitiser

Hand sanitiser
Ethanol 80% (or isopropyl alcohol 75%) Glycerol 1.45% Hydrogen peroxide 0.125% Water 18.43% (when using ethanol) or 24.43% (when using isopropyl alcohol)

*Adapted from World Health Organization guidance on approved hand rub formulations* ^1^

**Read more online**

**How to make 100 ml of hand sanitiser gel** **bit.ly/CEHJhandgel**
